# Evaluation of Nasal Mucociliary Clearance Using Saccharin Test Versus Charcoal Test Among Filipinos in a Tertiary Government Hospital

**DOI:** 10.7759/cureus.22065

**Published:** 2022-02-09

**Authors:** Rainier M Austero, January E Gelera

**Affiliations:** 1 Otolaryngology - Head and Neck Surgery, Amang Rodriguez Memorial Medical Center, Marikina, PHL

**Keywords:** filipino mucociliary, clearance test, mucociliary, saccharin, charcoal

## Abstract

Background: Nasal mucociliary clearance is mainly measured using the saccharin test because it is inexpensive, readily available, and non-toxic. However, in the local setting, the authors had difficulty procuring saccharin, and this prompted the authors to look for an alternative. Upon an expansive review of the literature, the authors came to know about the use of charcoal that has the same properties as saccharin plus it is inert and easily traceable. The objectives of this study were to (1) establish the normal nasal mucociliary clearance time (MCT) using the saccharin test (ST) and charcoal test (CT) among Filipinos, (2) determine if CT can be used to determine nasal mucociliary clearance and (3) determine if the age, sex, land of dwelling, and comorbidities can prolong MCT.

Methods: This is a cross-sectional study involving 50 subjects. ST and CT were performed by placing a particle of sodium saccharine and 10μg of charcoal on the medial surface and at least 1 cm behind the head of the inferior turbinate. All STs were done on the right nostril while CTs were done on the left. A 0- to 20-minute MCT was considered normal while MCT of more than 30 minutes was considered prolonged clearance. Lastly, a transit time of more than 60 minutes was considered a failed mucociliary clearance test.

Results: The mean mucociliary transit time for the saccharin group was 14.48 minutes while for the charcoal group was 14.78 minutes (p=0.531). When grouped into subcategories, CT results showed a higher mucociliary clearance mean time among males, provincial residents, and those without comorbidities while ST had a higher mean mucociliary clearance time among females, Metro Manila residents, and those with comorbidities. All were noted to be not statistically significant.

Conclusion: This study showed that CT is comparable with ST. Also, it can be used as an alternative to ST because the tester directly observes the charcoal transit in the oropharynx while ST relies on the patient's perception of taste that could result in false results.

## Introduction

Saccharin tests (STs) have been widely used to measure the nasal mucociliary clearance time (MCT) as they are inexpensive, readily available, and non-toxic [1]. However, saccharin is not easily procured in most countries and territories especially in our local setting, thus limiting the performance of these tests in patients who are suspected to have problems in nasal mucociliary clearance. Hence, various physicians continuously search for possible alternative agents that can parallel the properties of saccharin as a test agent in measuring MCT. Upon an expansive review of the literature, the authors came upon an article published in 1984 by Passàli et al. where vegetable charcoal powder mixed with saccharin was used to produce a solution to address the sol-gel layer of the mucociliary transport system [2]. The authors of this study decided to use charcoal as a single agent to test the MCT because of the belief that under normal conditions, the mucociliary transport system functions as a single unit; hence, it does not matter if the particulate used is water soluble or not [3]. Furthermore, this study compared the behavior of charcoal when used as an agent for MCT to saccharin. Lastly, the study determined whether nasal mucociliary function alters in a given condition such as age, sex, current health status, land of dwelling, and exposure to passive smoking.

## Materials and methods

This was a cross-sectional study conducted at a tertiary government training hospital, involving patients in the Otorhinolaryngology - Head and Neck Surgery outpatient department who underwent mucociliary clearance determination from January to May 2019. The institutional review board of ‘Amang’ Rodriguez Memorial Medical Center approved this study (IRB no. 2019-01-00).

Patients without any nasal complaints or conditions such as rhinorrhea or nasal obstruction were consecutively recruited and enrolled in the study. Those with chronic or terminal illness or with known impaired mucociliary clearance were excluded. The sample size was computed based on the power of the t-test with a confidence interval of 95% wherein the minimum number of patients to be included in the study was 49. In this study, the authors carried out the CT and ST in 50 subjects.

Information about the subject’s age, sex, smoking habits, and residence was recorded. The techniques for measuring the MCT were adapted from the studies of Anderson and Proctor [4] and Deborah and Prathibha [5,6]. Before the start of the test process, the subjects were placed in a stable environment where they were comfortable for at least an hour. Likewise, the subjects were instructed not to sniff, sneeze, cough, smoke, drink or eat during the test period. The MCT was measured from 8:00 am to 4:00 pm to eliminate the influence of circadian rhythm [4]. An MCT of 0-20 minutes was considered normal. The test was terminated after 60 minutes. The principal investigator performed all tests throughout the study period.

Saccharine test

A sodium saccharine particle, at least 1 mm across, was placed under direct vision on the medial surface and at least 1 cm behind the head of the inferior nasal turbinate. No pre-taste verification was done because saccharin taste may remain up to four hours that may alter the results of the study [4]. All STs were performed in the right nostril. The participants were instructed to be seated with their head tipped slightly forward during the whole procedure. They were further instructed to report if they noted any bitter-sweet taste. The time elapsed was recorded to the nearest minute and the test was considered complete. If the sweet taste was not appreciated after 60 minutes, then a saccharine particle was placed on the tongue for verification.

Charcoal test

In total, 10 μg charcoal powder was placed under direct vision, on the medial surface, and at least 1 cm behind the head of the inferior nasal turbinate. All CTs were done in the left nostril of the subjects. The participants were instructed to be seated with their head tipped slightly forward during the whole procedure. Every 30 seconds, the investigator would check for blackish coloration of the posterior pharyngeal wall. The time elapse was then recorded.

## Results

The study population age ranged from 19 to 77 years with a mean age of 36.6 ± 14.1 years. The majority (52%) of patients were female, non-smokers (74%), without comorbidities (80%), and residents within Metro Manila (52%). Among those subjects with comorbidities, 10 had hypertension (8%) (Table 1). The ST MCT results ranged from 3.17 to 44.76 minutes with an average of 14.48 minutes; 84% of the clearance time fell within the normal range. The CT nasal MCT ranged from 3.10 to 49.67 minutes with an average clearance time of 14.78 minutes. Also, 82% fell within the normal range. When the elapsed time was compared between ST and CT, no significant difference was noted (Table 2).

**Table 1:** Demographic characteristics of patients

Characteristic (N=50)	Percent/mean ± SD
Age (years)	36.6 ± 14.1
Sex	
Female	52.0
Male	48.0
Residence	
Metro Manila	52.0
Province	48.0
Smoking history	
No	74.0
Previously	6.0
Yes	20.0
Comorbidities	
No	80.0
Yes	20.0
Hypertension	8.0
Allergic rhinitis	4.0
Polycystic ovary syndrome	4.0
Bipolar disorder	2.0
Bronchial asthma	2.0
Pulmonary tuberculosis	2.0
Chronic rhinusinusitis	2.0

**Table 2:** Nasal mucociliary clearance results for saccharin test versus charcoal test p=0.05 (significant)

Saccharin test			Charcoal test		
Nasal mucociliary clearance time (N=50)	Percent/mean ± SD	p-value	Nasal mucociliary clearance time (N=50)	Percent/mean ± SD	p-value
Saccharin test (min)	14.48 ± 8.90	0.531	Charcoal test (mins)	14.78 ± 9.66	0.531
Normal (0-20 min)	84.0		Normal (0-20 min)	82.0	
Prolonged (21-30 min)	10.0		Prolonged (21-30 min)	10.0	
Severely prolonged (31-60 min)	6.0		Severely prolonged (31-60 min)	8.0	

Relationships between the nasal MCT results, both in ST and CT, and demographic characteristics (gender, location, and smoking history) and presence of comorbidities, were not significant (Table 3). Similarly, CT had a higher mean nasal MCT among males (17.30 minutes, SD ±9.96), provincial residents (15.78 minutes, SD ±11.38), non-smokers (15.30 minutes, SD ±10.93), and those without comorbidities (14.69 minutes, SD ±9.28). On the other hand, ST had a higher mean nasal MCT among females (12.89 minutes, SD ±8.32), Metro Manila residents (14.42 minutes, SD ±7.32), and those with comorbidities (14.85 minutes, SD ±9.48). A comparison of CT and ST results among gender, location of residences, smoking history, and presence of comorbidities also revealed no significant differences (Table 4). However, when the correlation of results with age was determined, it was noted that as the patient aged, the nasal MCT was longer for both tests, CT (p=0.021) and ST (p=0.010) (Table 5).

**Table 3:** Mucociliary transport time results for saccharin and charcoal tests based on patients’ profile p=0.05 (significant)

Mucociliary transport time (N=50)	Type of test (mean ± SD)		Mean difference	p-value
	Saccharin	Charcoal		
All patients	14.48 ± 8.90	14.78 ± 9.66	-0.2954	0.531
Among females	12.89 ± 8.32	12.45 ± 8.93	0.4361	0.474
Among males	16.21 ± 9.35	17.30 ± 9.96	-1.0879	0.137
Among Manila residents	14.42 ± 7.32	13.86 ± 7.86	0.5581	0.331
Among provincial residents	14.56 ± 10.51	15.78 ± 11.38	-1.2200	0.107
Among non-smokers	14.97 ± 9.97	15.30 ± 10.93	-0.3332	0.573
Among smokers	13.10 ± 4.74	13.29 ± 4.35	-0.1877	0.798
Among those with comorbidities	14.85 ± 9.48	14.80 ± 9.86	0.0487	0.908
Among those without comorbidities	13.02 ± 6.27	14.69 ± 9.28	-1.6720	0.330

**Table 4:** Comparison of saccharin and charcoal test results based on patient characteristics p=0.05 (significant)

p-value	Mean difference	Saccharin test	Mucociliary transport time (N=50)	Charcoal test	Mean difference	p-value
0.189	-3.3293	12.89 ± 8.32	Female	12.45 ± 8.93	-4.8533	0.075
		16.21 ± 9.35	Male	17.30 ± 9.96		
0.956	-0.1410	14.42 ± 7.32	Manila	13.86 ± 7.86	-1.9190	0.488
		14.56 ± 10.51	Province residents	15.78 ± 11.38		
0.520	1.8726	14.97 ± 9.97	Non-smokers	15.30 ± 10.93	2.0182	0.522
		13.10 ± 4.74	Smokers	13.29 ± 4.35		
0.567	1.8265	14.85 ± 9.48	Those with comorbidities	14.80 ± 9.86	0.1057	0.976
		13.02 ± 6.27	Those without comorbidities	14.69 ± 9.28		

**Table 5:** Correlation analysis for age versus nasal mucociliary clearance time *Statistically significant p=0.05 (significant)

Age (N=50)	Nasal mucociliary transport time	
	Saccharin test	Charcoal test
Correlation coefficient	0.363	0.327
p-value	0.010*	0.021*

## Discussion

Mucociliary clearance is an important aspect of the upper airway and sinus defense mechanism. It removes noxious inhaled material and pathogens by transporting the mucus, together with substances trapped in it, using the mucociliary escalator [7]. The normal mucociliary transit time in humans has been reported to be 12-15 minutes. The transit time of more than 30 minutes is considered to be abnormal and is indicative of impaired mucociliary clearance [8]. There are several methods used to evaluate the mucociliary clearance either directly using microscopic studies or indirectly by assessing the mucous transport or clearance. One study used a simpler method that consisted of depositing a small particle of saccharin on the nasal mucosa and noting the time that it took the subject to report a sweet taste [8]. The saccharin test is widely used in determining the nasal MCT because it is inexpensive, non-invasive, and simple to perform [9]. However, when the authors tried to replicate its use in the local settings, they encountered difficulty in procuring the agent, and hence they came upon trying out charcoal as an alternative to saccharin. In this study, the MCT of subjects using both saccharin and charcoal was determined. The study showed nasal MCT for the saccharin test and the charcoal test to be comparable (p=0.531). CT does not rely on taste, but rather direct observation of charcoal in the pharyngeal area. Also, activated charcoal is more widely available in the local pharmacy than saccharin; hence, CT can indeed be used as an alternative to ST.

The results also showed that as the patient ages, the MCT also increases, which supports the widely known notion that nasal mucosal lining atrophies with age. Another study found that older individuals have prolonged MCT probably due to the development of different comorbidities as an individual ages [10].

Furthermore, the study revealed that smokers have higher clearance time than non-smokers; however, this was not statistically significant (p=0.522). A local study showed otherwise, that nasal MCT is prolonged among smokers compared to the non-smokers [11]. However, some studies suggest that the effect of smoking on nasal MCT is temporary and mucociliary clearance may revert to its normal function after a certain period of smoking cessation, which could explain the results of the present study [11-14].

The limitation of this study was that it was conducted in the afternoon (around 2:00 pm) until the out-patient clinic closing time (5:00 pm). Performing the test during peak hours may be affected by the circadian rhythm of the body; thus, ideally MCT should be measured around 2:00 to 4:00 am [4]. Other factors such as pregnancy where hormonal changes affect the MCT should have been considered [15-17].

## Conclusions

This study showed that the charcoal test is comparable with the saccharin test when used to evaluate mucociliary clearance. Additionally, it is more reliable because the examiner can directly visualize the charcoal transit in the oropharynx. ST relies heavily on the subjective perception of taste that can be confounded by patients’ inattention or poor understanding of what to expect or what to observe, and thus can contribute to false or inaccurate results. This study also revealed that increasing age has an association with prolonged MCT, which strengthens the widely known notion that nasal mucosal lining atrophies with age. Further studies on the use of charcoal tests are recommended, especially in much bigger study groups to support the result of this study and for their future utilization as they have the potential to be useful among difficult patients, like in the pediatric age group, with special needs, and those with difficulty in verbal communication because charcoal can be easily traced by the examiner.
